# The time burden of overweight and obesity in primary care

**DOI:** 10.1186/1472-6963-11-191

**Published:** 2011-08-17

**Authors:** Adam G Tsai, Elmer D Abbo, Lorraine G Ogden

**Affiliations:** 1 Division of General Internal Medicine and Center for Human Nutrition, University of Colorado School of Medicine. Address: Campus Box C-263, 13001 E. 17th Place, Aurora, Colorado 80045, USA; 2 Section of Hospital Medicine, Pritzker School of Medicine, University of Chicago. Address: Mail Code 6098, 5841 S. Maryland Avenue, Chicago, Illinois 60637, USA; 3 Department of Biostatistics, Colorado School of Public Health, and Center for Human Nutrition, University of Colorado School of Medicine. Address: Campus Box C-263, 13001 E. 17th Place, Aurora, Colorado 80045, USA

## Abstract

**Background:**

Overweight and obesity are associated with many conditions treated in primary care. Our objectives were: 1) to determine the frequency of weight-related conditions in a national sample of outpatient visits in the United States; 2) to establish the percentage of diagnosis codes and visit codes attributable to overweight and obesity; and 3) to estimate time spent to address these conditions, including time attributable to overweight and obesity itself.

**Methods:**

We analyzed primary care visits from the 2005 and 2006 National Ambulatory Medical Care Survey (NAMCS) in the United States. Weight-related conditions included diabetes, hypertension, hyperlipidemia, obesity, cardiovascular disease, osteoarthritis, and low back pain. We used multivariable logistic regression to estimate an odds ratio for each weight-related condition, which we then converted to an attributable fraction (AF). The AF represents the percentage of diagnosis codes and visit codes attributable to excess weight for that condition. We then divided total visit time among all diagnoses and clinical items addressed at the primary care visit. Finally, to calculate the time attributable to overweight and obesity, we multiplied the AFs by the time spent on each weight-related condition.

**Results:**

The total number of clinical items (diagnoses + medications + tests + counseling) was estimated to be 7.6 per patient, of which 2.2 were weight-related. Of a total visit time of 21.77 minutes, time spent addressing weight-related conditions was 5.65 minutes (30%), including 1.75 minutes (8.0%) attributable to overweight and obesity.

**Conclusions:**

Approximately 8% of time from primary care visits is attributable to overweight and obesity. This estimate is conservative because the NAMCS only allows for coding of three diagnoses addressed per visit. Estimates of the time burden of overweight and obesity provide data to prioritize weight management for prevention and treatment.

## Background

Excess weight (overweight and obesity) is associated with an enormous burden of illness and is a root cause for many conditions seen by primary care providers (PCPs) [1,2]. For example, in the United States in 2005, hypertension accounted for 28.1% of all office visits, arthritis for 17.5%, diabetes for 11.9%, and depression for 10.4% [3]. In spite of the importance of overweight/obesity as a root cause for multiple medical problems, PCPs are not consistently able to address weight management in all eligible patients. Barriers to the provision of obesity treatment include the increasing complexity of primary care visits [4-6] as well as limitations in time, resources, training, and reimbursement [7-10].

PCPs clearly spend a significant portion of their time diagnosing and treating conditions related to excess weight. However, the time requirement has not been quantified. Previous studies have estimated the burden of obesity as the proportion of cases of a disease (e.g., diabetes) due to obesity or have estimated the percentage of health care spending attributable to obesity [11-14]. No study, however, has quantified the burden of time faced by primary care clinicians. In the context of a shortage of PCPs in the United States [15] and of higher visit complexity, time spent on a prevalent condition (obesity) is of great importance to clinicians, researchers, and policy makers.

The aims of this study were: 1) to estimate the number and prevalence of weight-related conditions in a nationally representative sample of outpatient visits in the United States; 2) to establish the percentage of diagnosis codes and visit codes attributable to overweight and obesity; and 3) to estimate the amount of time spent addressing these conditions. Our ultimate objective was to use the output from aims 2) and 3) above to estimate the amount of primary care visit time attributable to overweight and to obesity.

## Methods

The National Ambulatory Medical Care Survey (NAMCS) is a nationally representative sample of outpatient visits to physicians in the United States. NAMCS uses a multi-stage sampling strategy, with providers sampled randomly within defined geographic areas, known as primary sampling units (PSUs). A PSU is defined by NAMCS as a county or group of counties, a county equivalent (parish or independent city), a town or township, or a metropolitan statistical area. Clinicians and practice administrative staff enter information on a one-page data collection form that includes: 1) up to three diagnoses addressed at the visit; 2) chronic conditions, whether addressed at the visit or not; 3) medications prescribed; 4) tests ordered; 5) non-medication treatments recommended; and 6) counseling provided. Height and weight are also collected on this form and used to calculate body mass index (BMI). The data collection form also includes patient socio-demographics (age, gender, race and ethnicity, and insurance payer) and a field for "time spent with provider". NAMCS includes visits to both generalist and specialist physicians. The current analysis focused on visits to adult primary care physicians. These included visits listed under general internal medicine, family medicine, geriatrics, and general practice. All data in NAMCS are de-identified. Thus, the study was exempted from review by the Colorado Multiple Institutional Review Board.

We defined the following diagnoses/diagnostic categories as weight-related: diabetes (type 2), hypertension; hyperlipidemia; obesity; cardiovascular disease; depression; and musculoskeletal pain. Due to small sample sizes for some diagnosis codes, the category of cardiovascular disease was a composite of coronary artery disease, cerebrovascular disease, peripheral arterial disease, chronic kidney disease, and congestive heart failure. Similarly, musculoskeletal pain was a composite of osteoarthritis and low back pain. Because our goal was to divide visit time among the diagnoses and other clinical items addressed at the visit, we used only the field "diagnoses related to this visit." We did not use the field for chronic conditions ("Regardless of the diagnoses written in [the previous section], does the patient now have..."). We did not use this latter field because we could not be certain whether these conditions were addressed at the visit.

In addition to diagnosis codes, we counted subjects as having received treatment for weight-related conditions if a diagnosis was not coded but a medication was prescribed at the visit. For example, if a diabetes medication was prescribed but the diagnosis was not coded, we counted diabetes as a secondary diagnosis. Finally, we counted relevant diagnostic tests (e.g., hemoglobin A1c test for diabetes) and counseling codes (e.g., weight reduction counseling) under their respective conditions if they were ordered/conducted at the visit. An appendix, available on request from the first author, lists the diagnoses, medications, tests, and counseling fields that we included as weight-related.

A multivariable logistic regression model was constructed for each weight-related condition to estimate the odds of receiving treatment for the condition (indicated by a diagnosis code and/or a medication prescribed) by category of body mass index (BMI). BMI was categorized as normal weight (18.5-24.9 kg/m^2^), overweight (25-29.9 kg/m^2^), or obese (≥ 30 kg/m^2^) and entered into the logistic regression as a categorical variable. Individuals classified as underweight (BMI < 18.5 kg/m^2^) were not included because of potential confounding by medical illness. Regressions also controlled for patient age as a continuous variable, and for gender, race/ethnicity, insurance payer (as a proxy for socioeconomic status), number of previous visits during the past 12 months, and practice type (solo versus group) as categorical variables. Additional logistic models were constructed to estimate the odds of receiving each weight-related diagnostic test or counseling item by BMI category. The odds of receiving diagnostic tests and counseling codes were estimated separately from the diagnosis codes and medications because their association with overweight and obesity was substantially weaker. The SAS/STAT^® ^procedure SURVEYLOGISTIC was used to perform all analyses to include the NAMCS sample weights and to account for the stratification and clustering of the NAMCS sample design.

The logistic regression models were used to calculate an attributable fraction (AF) separately for each weight-related condition (diagnosis or medication), each weight-related diagnostic test, and each weight-related counseling code addressed at the office visit. The AF for each of these clinical items [16] was calculated as:

$$OAF=1-\frac{\sum_{j=1}^{n}w_{j}p\left(x_{0j},\hat{\beta }\right)}{\sum_{j=1}^{n}w_{j}p\left(x_{j},\hat{\beta }\right)},$$

where *n *is the number of patients, *w_j _*is the NAMCS sample weight for the *j*th patient, $p\left(x_{0j},\hat{\beta }\right)$ is the model-based predicted probability of the clinical item for patient *j *given his or her covariates but assuming the individual was normal weight, and $p\left(x_{j},\hat{\beta }\right)$ is the predicted probability of the clinical item for each patient given his or her covariates and observed obesity status. The interpretation of the AF in this study is the following: the percentage of weight-related clinical items (diagnosis/medication; diagnostic test; or counseling) *that can be attributed to overweight and obesity*. This definition is similar to that used in other epidemiologic analyses, in which the population attributable fraction, or PAF, is most commonly interpreted as the percentage of cases of a condition attributable to a given risk factor [17]. One difference between standard estimates of attributable risk and estimates from the current study is that the former use population-based samples, whereas estimates in this study are derived from a clinical population. A second difference is that, until recently, population attributable fractions in the obesity literature were calculated using a formula that included only two variables: the prevalence of the risk factor (e.g., obesity) and the relative risk of a disease (e.g., diabetes) among obese individuals, as compared to normal weight individuals [17]. This older formula does not control for possible confounding by other patient/subject characteristics. By conducting multivariable regression and converting the odds ratios to attributable fractions, we believed our estimates would be more valid.

We summed the total number of clinical items addressed at the office visit (including both weight-related and non weight-related diagnoses + medications + tests + counseling), following a slightly adapted version of published methods that have been previously applied to NAMCS data [4]. To divide visit time among these clinical items, we used the results of an empirical study of recorded physician visits [18]. In that analysis, half the time was spent addressing the primary diagnosis, and the remaining visit time was split among all other clinical items discussed at the visit. We followed this method, assigning half the time from the "Time Spent with Provider" field in the NAMCS to the primary diagnosis, and allocating the remaining time equally among all secondary diagnoses and other clinical items.

The allocation of time described above provided an estimate of the *total time spent *on each diagnosis or clinical item. The last step in the analysis was to calculate the *time attributable to overweight and obesity*. To do this, we multiplied the AFs (calculated from the logistic regression models) by the total time spent on each weight-related diagnosis/medication, test, or counseling item. We then summed these times within each condition (e.g., time spent addressing a diagnosis of diabetes + time spent prescribing medication for diabetes + time spent ordering a hemoglobin A1c test).

## Results

Table 1 shows the socio-demographic characteristics of the study sample, stratified by BMI category. A total of 1,745 (26.7%) patients were normal weight, 2011 (30.7%) were overweight, and 2612 (39.9%) were obese. An additional 181 (2.8%) individuals were underweight (BMI < 18.5 kg/m^2^) and were excluded from further analysis. Women comprised the majority of the sample and, relative to men, were more likely to be normal weight or obese and less likely to be overweight. The percentage of whites was lowest in the obese category and the percentages of African-Americans and Latinos were highest in the obese category.

**Table 1:** Sample demographics of combined 2005-2006 NAMCS sample, excluding BMI < 18.5 kg/m^2^*

BMI category†	Normal weight (n = 1745)	Overweight (n = 2011)	Obese (n = 2612)
**Age (mean ± SD)**	**50.7 ± 20.0^a^**	**52.8 ± 17.3^b^**	**51.1 ± 15.3^c^**

Body mass index, kg/m^2 ^(mean ± SD)	22.4 ± 1.7^a^	27.4 ± 1.5^b^	36.7 ± 6.6^c^
Gender (%)			
Female	64.7%^a^	52.3%^b^	58.7%^c^
Male	35.3%^a^	47.7%^b^	41.3%^c^
Race/Ethnicity (%)			
Non-Hispanic White	69.9%^a^	71.5%^a^	65.8%^b^
Non-Hispanic Black	11.6%^a^	11.5%^a^	15.7%^b^
Hispanic	9.7%^ab^	10.8%^b^	12.3%^bc^
Other or multi-racial	8.9%^a^	6.1%^b^	6.2%^b^
Insurance payer (%)			
Private insurance	47.3%^a^	46.6%^a^	46.9%^a^
Medicare	22.8%^a^	22.2%^a^	18.0%^b^
Medicaid	13.8%^a^	12.8%^ab^	15.0%^ac^
Self-pay	6.5%^ab^	7.9%^b^	8.9%^bc^
Other/unknown	9.7%^a^	10.5%^a^	11.3%^a^
Time with Provider (mean ± SD)	21.3 ± 11.6^a^	22.0 ± 13.6^a^	21.4 ± 12.4^a^

* For pairwise comparisons: columns with different superscript are significantly different from each other. T tests were used for continuous variables (PROC SURVEY MEANS) and Rao-Scott Chi-Square tests (PROC SURVEYFREQ) for categorical variables.

† Normal weight = 18.5-24.9 kg/m^2^; overweight = 25-29.9 kg/m^2^; obese = ≥ 30 kg/m^2^.

Table 2 lists the weight-related conditions included in the analysis, the percent of the NAMCS sample that had these diagnoses coded, and the estimated frequency of office visits for these conditions in the United States. Hypertension was coded nearly twice as commonly (28.2% of visits) as the next nearest diagnoses, hyperlipidemia (16.7%) and musculoskeletal disorders (15.4%). Diabetes was coded in 12.5% of visits, cardiovascular disease and depression in 9-10%, and obesity in 3.5%. It is estimated that over half (58.5%) of all adult patients attending an outpatient office visit were evaluated or treated for one of these weight-related conditions. The total number of clinical items per patient (diagnoses + medications + tests + counseling) was estimated (mean ± SE) to be 7.6 ± 0.11, of which 2.2 ± 0.11 were weight-related items. The number of clinical items and of weight-related clinical items increased with increasing BMI category (normal weight, overweight, obese), particularly among the obese (7.1 ± 0.3, 7.2 ± 0.3, and 8.2 ± 0.2 respectively for all clinical items, p < 0.0001; 1.5 ± 0.1, 1.9 ± 0.1, and 2.8 ± 0.15 respectively for weight-related items, p < 0.0001).

**Table 2:** Number of cases, sample percentage, and estimated population percentage of patients receiving each diagnosis or medication during the office visit (BMI recorded at office visit and excluding BMI < 18.5 kg/m^2 ^[N = 6,368])

Diagnosis or Medication	Number of Cases	% of Sample	Estimated % of Patient Population (95% CI)
Hypertension	1,797	28.22	32.02 (28.55, 35.49)
Diabetes	793	12.45	11.73 (10.05, 13.41)
Hyperlipidemia	1,064	16.71	19.93 (17.15, 22.71)
*Musculoskeletal	978	15.36	20.83 (17.51, 24.14)
†Cardiovascular disease	617	9.69	12.48 (10.21, 14.76)
Depression	578	9.08	9.61 (7.82, 11.41)
‡Obesity	225	3.53	3.03 (2.12, 3.94)
Any above diagnosis or medication	3,473	54.54	58.44 (54.37, 62.53)

*Musculoskeletal: a composite diagnosis that included low back pain and osteoarthritis

†Cardiovascular disease: a composite diagnosis that included coronary artery disease, cerebrovascular disease, peripheral arterial disease, chronic kidney disease, and congestive heart failure.

‡Obesity numbers and percentages based on diagnosis codes only.

Table 3 shows the odds ratios for a diagnosis or medication being coded at the visit based on the presence of overweight/obesity, as well as the corresponding AF. Nearly 57% of diabetes diagnoses and/or medications were attributable to overweight/obesity, as were 23-28% of diagnoses and/or medications for hypertension, hyperlipidemia, and cardiovascular disease. Coding of depression was not significantly associated with overweight/obesity and thus fell out of additional analysis. Nearly 89% of coded obesity diagnoses were attributable to overweight or obesity. The percent of obesity diagnoses attributable to overweight/obesity was not 100%, possibly because: 1) a small number of patients were misclassified due to measurement error (misreporting of height and/or weight); or 2) the diagnosis was miscoded on the data collection sheet. Attributable fractions in the current analysis were consistent with previously published population attributable fractions for common weight-related conditions [14,19].

**Table 3:** Odds ratios and attributable fractions (AFs) for receiving a diagnosis code or having a medication prescribed during the office visit.*

Diagnosis or Medication	Number of Cases	Estimated Population %	Odds Ratio	p-value	AF
Diabetes					
Normal	105	5.14	1.00	< 0.0001	56.67%
Overweight	182	8.04	1.60 (1.09, 2.35)		
Obese	506	19.42	5.08 (3.57, 7.22)		
Hypertension					
Normal	350	22.84	1.00	< 0.0001	27.11%
Overweight	525	30.30	1.38 (1.08, 1.76)		
Obese	922	39.98	2.50 (1.98, 3.14)		
Hyperlipidemia					
Normal	214	14.14	1.00	< 0.0001	28.36%
Overweight	365	20.33	1.46 (1.12, 1.90)		
Obese	485	23.75	2.06 (1.55, 2.74)		
Musculoskeletal					
Normal	251	17.80	1.00	0.0005	13.10%
Overweight	296	19.71	1.07 (0.86, 1.34)		
Obese	431	23.90	1.46 (1.19, 1.80)		
Cardiovascular disease					
Normal	139	9.71	1.00	0.0001	23.13%
Overweight	201	12.97	1.33 (1.02, 1.73)		
Obese	277	14.07	1.78 (1.36, 2.34)		
Depression					
Normal	145	9.09	1.00		
Overweight	182	9.40	1.15 (0.88, 1.49)	0.468	8.59%
Obese	251	10.16	1.16 (0.90, 1.48)		
Obesity†			0, 1.00)		
Normal	10	0.40	1.00	< 0.0001	88.68%
Overweight	35	1.16	3.83 (1.28, 11.48)		
Obese	180	6.45	21.01 (5.92, 74.53)		

*Logistic regression models adjusted for age, gender, race/ethnicity, insurance status, type of practice (solo or group), and number of past visits during the previous 12 months. Regressions were limited to patients with BMI recorded at visit and excluded those with BMI < 18.5 kg/m^2^.

†Obesity odds ratios and AFs based on diagnosis codes only.

Table 4 shows the odds ratios and corresponding AFs for diagnostic tests and counseling codes performed at the visit. As noted above, the AFs for these codes were smaller than those for diagnoses and medications (i.e., a weaker association between overweight/obesity and weight-related diagnostic tests or counseling codes). For example, the AF for blood pressure screening was negligible, even though the AF for a diagnosis or medication for hypertension (Table 3) was 27.1%. This is because all patients presenting for primary care undergo blood pressure measurement, regardless of weight. A similar analogy applies for cholesterol screening, which carried a low AF. Diagnostic tests and counseling codes specific to weight, however (e.g., weight reduction counseling), had a high AF, in the same range as the AFs for coded diagnoses and medications.

**Table 4:** Odds ratios and obesity-attributable fractions (AFs) for clinical items*

Diagnosis	Number of Cases	Estimated Population %	Odds Ratio	p-value	AF (overweight + obese)
HbA1c					
Normal	68	3.71	1.00	< 0.0001	38.72%
Overweight	94	4.27	1.09 (0.75, 1.58)		
Obese	251	9.29	2.78 (1.93, 4.01)		
Blood Pressure					
Normal	1,704	97.15	1.00	0.569	0.05%
Overweight	1,957	96.91	0.92 (0.53, 1.58)		
Obese	2,558	97.55	1.14 (0.69, 1.88)		
Cholesterol					
Normal	296	18.98	1.00	0.003	7.08%
Overweight	374	18.73	0.93 (0.75, 1.16)		
Obese	540	23.08	1.33 (1.04, 1.69)		
Diet/Nutrition					
Normal	284	16.98	1.00	< 0.0001	23.04%
Overweight	404	19.09	1.15 (0.91, 1.44)		
Obese	759	28.26	1.93 (1.55, 2.41)		
Exercise					
Normal	228	14.57	1.00	< 0.0001	23.61%
Overweight	327	17.12	1.22 (0.96, 1.54)		
Obese	606	23.66	1.85 (1.41, 2.43)		
Weight Reduction					
Normal	26	1.3	1.00	< 0.0001	87.54%
Overweight	117	6.22	5.55 (2.51, 12.28)		
Obese	496	19.22	19.27 (8.55, 43.44)		
Depression					
Normal	84	6.05	1.00	0.245	-24.58%
Overweight	78	4.06	0.73 (0.44, 1.21)		
Obese	78	3.79	0.68 (0.42, 1.09)		
EKG					
Normal	81	6.86	1.00	0.430	-13.53%
Overweight	106	6.05	0.84 (0.55, 1.29)		
Obese	113	5.26	0.80 (0.57, 1.13)		
Physical Therapy					
Normal	37	2.38	1.00	0.756	-6.71%
Overweight	37	2.37	0.99 (0.65, 1.50)		
Obese	53	2.17	0.85 (0.54, 1.35)		

*Regressions adjusted for age, gender, race/ethnicity, insurance status, solo vs. group practice, and number of visits in the previous 12 months. Regressions limited to patients with recorded BMI, excluding BMI < 18.5 kg/m^2^.

**Figure 1:**
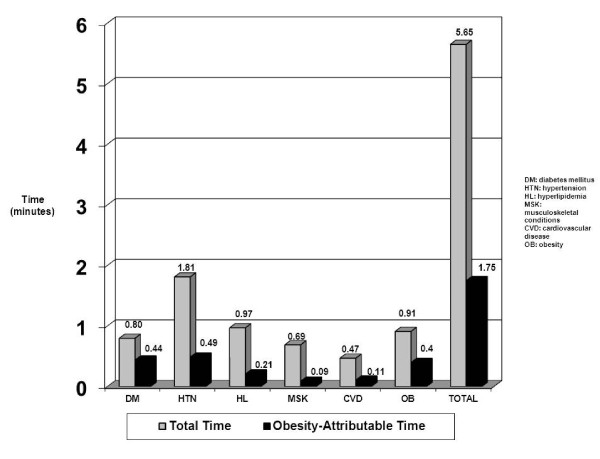
**Physician visit time per condition and time per visit due to overweight/obesity**.

The Figure 1 shows the time spent per visit on each weight-related condition, including the time attributable to overweight/obesity for that condition. Time attributable to overweight/obesity for diabetes and hypertension both accounted for an average of 0.4-0.5 minutes per visit, and obesity accounted for an additional 0.4 minutes. The percentage of time attributable to overweight/obesity for each condition corresponded roughly to the AF listed in Table 3. The exception to this was obesity itself, for which the AF in Table 3 was 88.7%, but the percentage of time attributable was 44.4%. The lower percentage is likely explained by the inclusion of counseling codes (under the diagnosis category of obesity) that are more common for overweight and obese patients but are not limited to these patients. For example, the AFs for diet/nutrition counseling and for exercise counseling were 23.0% and 23.6%, respectively.

The total time spent on all weight-related conditions was 5.65 minutes per visit, of which 1.75 minutes were *attributable to overweight and obesity *(see Figure 1). These 1.75 minutes accounted for 8.0% of the total mean visit time in the NAMCS of 21.77 minutes - equal to 38 minutes out of a hypothetical 8-hour work day. In a secondary analysis where all patient visits in the sample were used to estimate the time spent treating each condition (regardless of whether BMI was recorded), overall results were similar - 40 minutes out of an 8-hour work day were spent treating weight-related diagnoses and clinical items (data not shown). Finally, in a secondary analysis accounting for only the effect of obesity (BMI ≥ 30 kg/m^2^), total time attributable to weight-related conditions was 23.5 minutes per day (4.9% of total visit time).

## Discussion

In this analysis of data from the NAMCS, we found that excess weight accounts for 38 minutes, or 8% of an 8-hour day for a primary care provider in the United States. The 8% represents the amount of time spent to evaluate and treat patients with weight-related conditions *that can be attributed to a single risk factor, excess weight (i.e., overweight and obesity)*. We consider this estimate to be conservative, for two reasons. First, we elected to include only the three diagnoses addressed at the visit but did not include other chronic conditions, some of which likely would have been evaluated or treated. Second, we could not account in this dataset for several other weight-related diagnoses (e.g., uterine, colon, and breast cancer, and gall bladder disease) because of inadequate sample sizes for multivariable analysis.

Although 8% is likely an underestimate, these results offer a qualitatively different way to assess how overweight and obesity affect primary care medical practice. The results suggest that, in a hypothetical situation where all patients have a BMI < 25 kg/m^2^, PCPs would have nearly 40 more minutes in their day. Forty minutes would be enough to see 2 additional patients, or alternatively, to spend a few more minutes with each patient to improve quality of care (e.g., to raise cancer screening rates). These results extend the work of Pearson et al, who used NAMCS data and found that visits for obese patients were slightly longer and involved a significantly greater number of medications prescribed [20]. The results also extend the work of Bertakis and Azari, who found in a study of directly observed visits that obesity was not significantly related to visit length, but that physicians conducted more technical tasks (e.g., medication prescribing) and less health education for their obese patients [21]. Our results confirm the findings in these two studies, and extend that work by estimating the total amount of physician time required for these tasks that can be attributed to overweight/obesity itself.

Attributable fractions have been used in previous research to assign a proportion of cases of a disease or a proportion of health care costs to obesity [14,19]. The unique aspects of this study are: 1) the combination of several methods that allowed us to estimate the burden of time attributable to a single risk factor (excess weight); and 2) the use of multivariable analysis to address potential confounding in previous estimates of attributable risk. The methods used in this study could be replicated to estimate the burden of other health risk factors (e.g., physical inactivity, alcohol or tobacco abuse) that are associated with multiple medical conditions.

This study has several limitations. First, only half of the NAMCS sample had both height and weight recorded on the data collection sheet, with the majority of missing data due to missing height measurements. Despite the limitations of BMI measurement, no significant difference was observed between the weights of those with and without height measured in the dataset (183.4 lbs vs 183.3 lbs; p = 0.91). Thus, the measurement of height (and the calculation of BMI) is not biased towards heavier or towards lighter individuals. NAMCS remains the best dataset available in the United States to analyze the characteristics of a nationally representative sample of primary care visits [22]. Second, small sample sizes for some conditions necessitated combining them. Attributable fractions (AFs) may not be identical for some conditions that were combined (e.g., low back pain and osteoarthritis). Third, our division of time among diagnoses and clinical items at the visit was based on a single empirical study of observed physician visits [18]. A study comparing NAMCS administrative data with information from directly observed visits reported that although PCPs in the United States overestimate visit duration, they also underestimate the number of services provided within a visit [23]. Fourth, these data are cross-sectional, and thus it is unknown whether the obesity preceded (or followed) the diagnosis codes used in the study.

## Conclusions

This analysis is the first to quantify the time burden of overweight and obesity. In a national sample of primary care visits in the United States, we found that overweight and obesity accounted for 8% of provider time. These results should be useful for clinicians, researchers, and policy makers considering resource intensity of weight loss interventions. For example, analyses of weight loss interventions could use these results as an estimate of provider time in a standard care group. More broadly, increased attention to weight management in clinical settings has been advocated as part of a multi-faceted approach to the obesity epidemic [24,25]. Studies such as this provide data to facilitate the implementation of treatment.

## Abbreviations

NAMCS: (National Ambulatory Medical Care Survey); AF: (attributable fraction); PCP: (primary care provider).

## Competing interests

The authors declare that they have no competing interests.

## Authors' contributions

AGT conceived of the overall study design, secured funding, assisted with the analysis, and led the drafting of the manuscript. EDA acquired the data, assisted with study design and data analysis, and edited the manuscript. LGO assisted with study design, led the data analysis, and edited the manuscript. All authors read and approved the final manuscript.

## Pre-publication history

The pre-publication history for this paper can be accessed here:

http://www.biomedcentral.com/1472-6963/11/191/prepub
